# Prevalence of cryptococcal meningitis and lymphadenitis among PLHA

**DOI:** 10.1186/1471-2334-14-S3-P77

**Published:** 2014-05-27

**Authors:** MV Narasimham, Pritilata Panda

**Affiliations:** 1 PG Department of Microbiology, MKCG Medical College & Hospital, Berhampur, India

## Background

Cryptococcosis has been a common cause of morbidity and mortality in immunocompromised patients. This study was conducted to estimate the prevalence of cryptococcosis in PLHA cases presenting mainly with meningitis and lymphadenitis.

## Methods

An ongoing prospective study conducted from June 2012- October 2013 in 90 HIV seropositive patients presenting with various complaints. CSF and lymphnode aspirates were subjected to preliminary microscopic examination, c (Giemsa staining, mucicarmine staining, negative staining and Gram’s staining). Latex agglutination test was done for CSF samples. Sampls inoculated on SDA at 37^0^C and 25^0^C followed by Bird seed agar (BSA). Identification was done based on demonstration of encapsulated budding yeast cells in microscopy, negative staining with Nigrosin, red capsule on mucicarmine, yeast like creamy mucoid colony on SDA at 37^0^C, brown to black colony on BSA, inositol assimilation and positive urease test.

## Results

Maximum cases were between 25-40 years of age. 4 meningitis & 2 lymphadenitis cases showed positive cryptococcal growth on microscopy and culture. Both CSF and lymph node aspirate were found to be positive in 1(1.11%) case. 2 cases of disseminated cryptococcosis were seen with one involving multiple lymph nodes & other was a case of meningitis & lymph node involvement.All the positive cryptococcal cases had a CD4 count <200 cells/µl.

## Conclusion

Cryptococcal infections without treatment have a high morbidity rate and appropriate systemic antifungal therapy can significantly improve the outcome. A high index of clinical suspicion and mycological surveillance is required to help in an early diagnosis & treatment.

